# Association of *APOBEC3G* genotypes and CD4 decline in Thai and Cambodian HIV-infected children with moderate immune deficiency

**DOI:** 10.1186/1742-6405-9-34

**Published:** 2012-11-24

**Authors:** Torsak Bunupuradah, Mayumi Imahashi, Thatri Iampornsin, Kazuhiro Matsuoka, Yasumasa Iwatani, Thanyawee Puthanakit, Jintanat Ananworanich, Jiratchaya Sophonphan, Apicha Mahanontharit, Tomoki Naoe, Saphonn Vonthanak, Praphan Phanuphak, Wataru Sugiura

**Affiliations:** 1 HIV-NAT, the Thai Red Cross AIDS Research Centre, 104 Ratchadamri Road, Pathumwan, Bangkok, 10330, Thailand; 2 Clinical Research Center, National Hospital Organization Nagoya Medical Center, Nagoya, Japan; 3 Program in Integrated Molecular Medicine, Graduate School of Medicine, Nagoya University, Nagoya, Japan; 4 Department of Pediatrics, Faculty of Medicine, Chulalongkorn University, Bangkok, Thailand; 5 Department of Medicine, Faculty of Medicine, Chulalongkorn University, Bangkok, Thailand; 6 SEARCH, the Thai Red Cross AIDS Research Centre, Bangkok, Thailand; 7 Social Health Clinic, Phnom Penh, Cambodia

**Keywords:** *APOBEC3G*, Treatment-naïve, HIV-infected children, Disease progression, PREDICT

## Abstract

**Introduction:**

Human *APOBEC3G* is a host defense factor that potently inhibits HIV replication. We hypothesize that HIV-infected children with a genetic variant of *APOBEC3G* will have a more rapid disease progression.

**Methods:**

Antiretroviral therapy (ART)-naïve children, aged 1–12 years old with CD4 15-24% and without severe HIV-related symptoms were enrolled. The children had CD4% and absolute CD4 counts every 12 weeks and HIV-RNA every 24 weeks until 144 weeks. ART was started when CD4% declined to < 15% or AIDS-related events developed.

*APOBEC3G* genetic variants were performed by PCR-based restriction fragment length polymorphism techniques from peripheral blood mononuclear cells. Random-effect linear regression analysis was performed to correlate APOBEC3G genotypes and disease progression.

**Results:**

147 children, 35% male, with a median (IQR) age of 6.5 (4.3-8.8) years were enrolled. CDC N:A:B were 1:63:36%. Median baseline values were 20% for CD4% 605 cells/mm^3^ for CD4 count and 4.7 log_10_copies/mL for HIV-RNA.

The frequencies of *APOBEC3G* genotypes AA (186H/H), AG (186H/R), GG (186R/R) were 86%, 12%, and 2% respectively. The *APOBEC3G* genotype GG was associated with a significant decline in CD4% -5.1% (−8.9 to −1.2%), p<0.001, and CD4 counts −226 (−415 to −34) cells/mm^3^, p<0.001 by random-effect liner regression analysis. No significant associations of *APOBEC3G* genotypes with HIV-RNA changes overtime (p=0.16) or progression to CDC B and C (p=0.49) were observed.

**Conclusions:**

*APOBEC3G* genotype GG was significantly associated with a more rapid decline in CD4. *APOBEC3G*’s antiviral effects on HIV disease progression in children should be further explored.

## Introduction

In recent years, one of the most investigated human genetic factors in the control of HIV-1 replication and disease progression is apolipoprotein B mRNA editing enzyme, catalytic polypeptide-like 3G (*APOBEC3G*) [1-4]. Human *APOBEC3G* is a host defense cellular factor located on chromosome 22q13.1-13.2. It interferes with the replication of HIV-1 in the absence of Vif, partly through a deaminase-dependent mechanism. The expression of *APOBEC3G* leads to excessive G (guanidine)-to-A (adenine) substitutions of viral cDNA, known as hypermutation. This destroys the coding and replicative capacity of the virus. As well as this, APOBEC3G inhibits HIV-1 reverse transcription, integration and restrict the infectivity of HIV-1 [1].

Previously, association of *APOBEC3G*, CD4 and HIV-RNA levels in HIV-infected adults has been reported. Higher levels of *APOBEC3G* mRNA were associated with lower plasma HIV-RNA in antiretroviral therapy (ART)-naïve HIV-infected adults [5,6]. Reddy K. et al. reported a genetic variant of *APOBEC3G*; H186R in GG, that was significantly associated with a decline in CD4 T cells and higher HIV-RNA among ART-naïve African HIV-infected adults [2]. However, there is only limited data of *APOBEC3G* in HIV-infected children [3].

We have conducted a multicentre, randomised, open-label trial of early ART. Compared to ART deferred until the CD4 percentage fell below 15% or the development of a CDC classification C event. This trial was conducted among ART-naïve HIV-infected children aged 1–12 years with CD4 15-24% (The PREDICT study, NCT00234091) [7,8]. Here, we report the effect of *APOBEC3G* genotypes on the disease progression rate in ART-naïve Thai and Cambodian HIV-infected children without AIDS symptoms and moderate immune deficiency in the PREDICT study.

## Methods

### Study population

This is a sub-study of the Pediatric Randomized Early versus Deferred Initiation in Cambodia and Thailand study (The PREDICT study, clinicaltrials.gov identification number NCT00234091). HIV-infected children were enrolled from 7 Thai and 2 Cambodian sites from March 2006 through September 2008. The inclusion criteria were ART-naïve HIV-infected children, aged 1–12 years, Center for Disease Control and Prevention (CDC) clinical classification N (no HIV symptoms) A (mild HIV symptoms) or B (moderate HIV symptoms) [9,10], CD4 15%-24%, hemoglobin ≥ 7.5 g/dL and no active opportunistic infections at screening visit. The children were followed by weight, height, physical examination, CD4%, CD4 count every 12 weeks and plasma HIV-RNA every 24 weeks until 144 weeks. ART was started when either the CD4% declined to < 15% or CDC classification C events developed. This study was approved by the local and the Ministry of Public Health Institutional Review Boards. All caregivers gave consent prior to the enrolments.

### Genotypes of APOBEC3G H186R polymorphism

In this study, we used the samples from all children in the deferred-arm of the PREDICT study. The *APOBEC3G* genotypes for three polymorphisms (186H/H, 186H/R and 186R/R) were determined by polymerase chain reaction (PCR)-Restriction Fragment Length Polymorphism (RFLP) described in previous reports [2,11]. The genomic DNA from peripheral blood mononuclear cell lysates in the PREDICT study were isolated using the Invisorb® Spin Blood Mini kit (Invitek, Berlin, Germany). The PCR reaction was performed to amplify 409-bp DNA fragment of *APOBEC3G* H186R region using the *APOBEC3G* forward primer: 5’-acctgtgggtctgctctgat-3’ and *APOBEC3G* reverse primer: 5’-caggagggaaggcaggag-3’. The PCR was performed in a total volume of 20 μL which contained 10 μl of 2x HotStarTaq Plus Master Mix (Qiagen Inc., Valencia, CA), 1μl of 10 μM of each primer and 80 ng of genomic DNA. PCR were started with denaturing at 95°C for 5 min, followed by 35 cycles of 94°C for 30 sec, 64°C for 30 sec and 72°C for 45 sec, with a final extension at 72°C for 7 min and 4°C. The PCR products were resolved by electrophoresis in 2% agarose gels in 1x TAE and were stained with ethidium bromide.

The PCR products were purified using QIA Cube MinElute kit (Qiagen Inc.). The restriction digestion with restriction endonucleases *Hha*I was conducted to determine the H186R genotype. The reaction was performed in a final volume of 10 μl with 37°C incubation for 2 hours and digested PCR products were separated in 2% agarose gel. The genotypes were reported as genotype AA (186H/H), AG (186H/R), or GG (186R/R)

### Statistical analysis

Variable distributions were described as median, interquartile range (IQR) or proportions as appropriate. Kruskal-Wallis test and the Chi tests were used for their comparisons of continuous covariates and categorical covariates, respectively. Random-effect linear regression analysis, adjusted by baseline and study week, was performed to assess the genetic effect of *APOBEC3G* genotypes on rates of disease progression overtime; CD4%, CD4 counts and plasma HIV-RNA. All significance tests were two-sided with significance at the 0.05 level. All analyses were undertaken using STATA 10.0 (StataCorp. 2007. Stata Statistical Software: Release 10. College Station, TX: StataCorp LP).

## Results

A total of 147 ART-naïve HIV-infected children, 35% male, with a median (IQR) age of 6.5 (4.3-8.8) years were enrolled. All children were infected with HIV via mother-to-child transmission. Majority (59%) were Thai and 41% were Cambodian. The median (IQR) baselines of CD4%, CD4 count and HIV RNA were 20 (17–23)%, 605 (460–846) cells/mm^3^ and 4.7 (4.3-5.0) log_10_copies/mL, respectively. The other baseline characteristics are shown in Table 1.

**Table 1:** **Baseline characteristics of antiretroviral therapy naïve 147 HIV**-**infected children**

**Characteristics**^**1**^	**Total (n**=**147)**	***APOBEC3G *subtype AA (n**=**127)**	***APOBEC3G *subtype AG (n**=**17)**	***APOBEC3G *subtype GG(n**=**3)**	**P value**
Age (years)	6.5 (4.3-8.8)	6.7 (4.3-8.8)	6.3 (4.2-7.9)	2.8 (1.2-10.1)	0.55
% male	35%	35%	35%	33%	0.99
% CDC clinical classification N:A:B	1:63:36%	1:67:32%	6:35:59%	0:33:67%	0.05
Weight (kg)	17 (13.5-21.5)	17 (14–21.5)	16 (13.5-21)	11.5 (8.6-21)	0.39
Height (cm)	108 (95–121)	110 (95–121)	103 (99–115)	86 (70–120)	0.39
Weight for age z-score	−1.32 (−2.03 to −0.79)	−1.27 (−2.02 to −0.77)	−1.33 (−2.03 to −0.89)	−1.66 (−2.11 to −1.32)	0.72
Height for age z-score	−1.66 (−2.5 to −0.93)	−1.66 (−2.48 to −0.97)	−1.39 (−2.33 to −0.87)	−2.72 (−3.01 to −1.83)	0.30
Weight for height z-score	−0.43 (−1.22 to 0.09)	−0.44 (−1.22 to 0.12)	−0.54 (−1.11 to −0.27)	0.03 (−0.4 to 0.37)	0.32
Baseline CD4%	20 (17–23)	20 (17–23)	19 (17–26.5)	22 (17–23)	0.81
Baseline CD4 count (cells/mm^3^)	605 (460–846)	601 (449–846)	620 (551–836)	1333 (675–2195)	0.09
Baseline HIV-RNA (log_10_copies/mL)	4.7 (4.3-5.0)	4.6 (4.3-5)	4.9 (4.4-5)	5 (3.9-5.5)	0.43

^**1**^Data are presented as median (IQR).

*APOBEC3G* genotypes AA (186H/H), AG (186H/R), GG (186R/R).

### APOBEC3G H186R polymorphism and genotypes

The frequencies of *APOBEC3G* genotypes AA (186H/H), AG (186H/R) and GG (186R/R) were 86% (n = 127), 12% (n = 17) and 2% (n = 3), respectively. The proportion of *APOBEC3G* genotypes AA:AG:GG in Thais were 84%:13%: 3% and in Cambodian were 90% :10% : 0%.

### Clinical progression and CD4 decline during 144 weeks

No death or loss to follow up was reported during baseline to week 144. Thirty children progressed to the CDC classification B (e.g. bacterial pneumonia, thrombocytopenia, herpes zoster, herpes simplex). Two girls progressed to CDC classification C; one girl had esophageal candidiasis (age at onset was 2.1 years, CD4 at onset was 22%) and another girl developed extrapulmonary tuberculosis (age at onset was 10 years, CD4 at onset was 27%) during their follow-ups.

A total of 69 children had started ART and 78 children were still ART-naïve at week 144. The reasons to started ART were worsening of clinical criteria in 3 children (1 *Pneumocystis jiroveci* pneumonia and 2 severe thrombocytopenia) and immunologic criteria in 66 children (63 for CD4 <15% and 3 for CD4 <20%). The median (IQR) CD4% and CD4 count at weeks 144 of those 69 children who started ART were 29 (24–34)% and 836 (620–1078) cells/mm^3^. On the other hand, the median (IQR) CD4% and CD4 count of 78 ART-naïve children at week 144 were 21 (17–25)% and 552 (436–711) cells/mm^3^.

### Association between APOBEC3G genotypes and clinical progression and CD4 decline

The proportion who started ART because of CD4 decline/clinical progression among children with *APOBEC3G* genotypes AA, AG, and GG were 60/127 (47%), 7/17 (41%) and 2/3 (67%) respectively (p=0.71). No significant association between the *APOBEC3G* genotypes and the CDC classification B/C was found (p=0.49).

**Figure 1:**
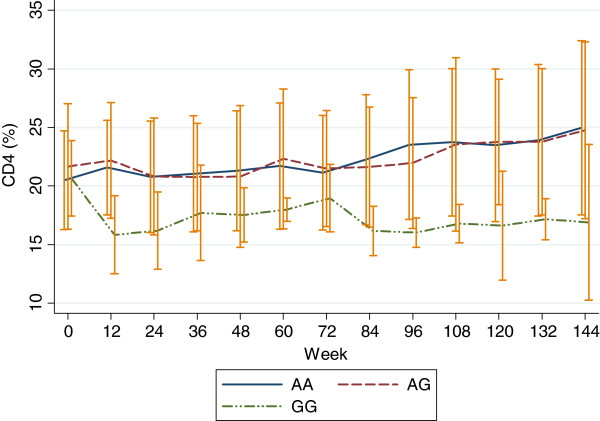
***APOBEC3G *subtypes and decline of CD4**% **over 144 weeks.**

By random-effect linear regression analysis, after adjustment by baseline CD4%, CD4 count and study week, it was demonstrated that the *APOBEC3G* genotype GG was associated with significant decline from baseline to week 144 in CD4% (95% confidence interval) -5.1% (−8.9 to −1.2%), p<0.001, and CD4 counts −226 (−415 to −34) cells/mm^3^, p<0.001. However, *APOBEC3G* genotype AG was not significantly associated with a change in CD4% and CD4 count over 144 weeks; CD4% (95% confidence interval) -0.2% (−1.9 to 1.5%), p=0.81, and CD4 counts 39 (−42 to 120) cells/mm^3^, p=0.35. Figure 1 shows the decline of CD4% over 144 weeks of each *APOBEC3G* genotypes. In addition, no significant association between the *APOBEC3G* genotypes and change in HIV-RNA log_10_ overtime was found (p=0.16).

## Discussion

In this study, we demonstrated the association of a genetic variant of *APOBEC3G* genotypes, H186 in GG, with the decline in CD4% and the CD4 count over time in Thai and Cambodian ART-naïve HIV-infected children with moderate immune deficiency. In contrast, no significant association of the *APOBEC3G* genotypes with changes of HIV-RNA log_10_ overtime or progression of CDC classification was found.

The frequency of *APOBEC3G* genotypes had been reported in HIV-infected children and had found to be an uncommon polymorphism (1% or less). De Maio FA et al. reported *APOBEC3G* genotypes in Argentinian perinatally HIV-infected children, AA, AG and GG were 81.8%, 17.9% and 0.3%, respectively [12]. In addition, *APOBEC3G* genotypes in Argentinian HIV-1-exposed uninfected children, AA, AG and GG were 85%, 14% and 1%, respectively [12]. The proportions of *APOBEC3G* genotypes in our study were comparable to that study [12].

*APOBEC3G* genotypes have been associated with significant decline in CD4 count in ART-naïve African HIV-infected adults [2]. In our study, we also found the association of *APOBEC3G* genotypes with significant decline in CD4% and CD4 count. This association needs more study in a larger sample size or other ethnicity as the mechanism is unclear [2] and warrants further investigation.

Association between the *APOBEC3G* genotypes and changes of HIV-RNA overtime was demonstrated in HIV-infected adults. Reddy et al. reported *APOBEC3G* genotype GG was associated with higher HIV-RNA compared to genotype AA (p=0.036) in African HIV-infected adults after primary HIV-1C infection [2]. However, we failed to demonstrate this association in our study which may be due to 1) different immune responses in children compared to adults and a different HIV-1 clade. The majority of Thais are infected by HIV subtypes CRF01_AE [13]. There is limited data of *APOBEC3G* genotypes and association to the changes in HIV-RNA in populations infected with different HIV clades. 2) Children enrolled in PREDICT study had lower median baseline HIV-RNA compared to HIV-infected children in general which had more AIDS symptoms [5 (4.9-5.5) log10 copies/ml] [14].

Our study had several limitations. First, very few children developed AIDS during the study, therefore, we could not find a significant correlation between *APOBEC3G* genotypes and the AIDS disease progression. A previous report in Argentinian HIV-infected children has also shown that there was no affect of *APOBEC3G* genotypes and AIDS onset [12]. In addition, only 3 children had *APOBEC3G* genotype GG, thus, the association of *APOBEC3G* genotypes and sharp decline of CD4% may have occurred by chance. The interpretation of our findings should be cautious as failure to demonstrate a statistically significant difference between study groups at baseline. For example, different of baseline CDC classification ratio, and higher baseline CD4 cell counts of subtype GG group than the other two groups, although the baseline CD4% was similar, does not ensure that the groups are equivalent, particularly with the small sample size of the subtype GG that limits statistical power. Second, we could not see a correlation of *APOBEC3G* genotypes with the clinical patterns of long term non-progressors, (defined as children who survived to 8 years or older with CD4% ≥ 25% without receiving ART [15,16]) because only children with CD4 <25% were enrolled in PREDICT study. Third, we were unable to perform *APOBEC3G* mRNA level as the method that we used could extract only DNA. Higher *APOBEC3G* mRNA level has been associated with higher CD4 count and lower HIV-RNA levels in ART-naïve HIV-infected adults [5,6].

The strengths of our study are the large number of ART-naïve vertically transmitted HIV-infected children with moderate immune deficiency in Asia enrolled with the long-term clinical data with 144-weeks of follow-up. Further studies should be evaluated for better understanding of APOBEC3G’s antiviral effects on the disease progression in the HIV-infected children.

## Conclusions

In conclusion, our data showed that a genetic variant of *APOBEC3G* genotypes, H186 in GG, was significantly associated with decline in CD4% and the CD4 count over time in Thai and Cambodian ART-naïve HIV-infected children with moderate immune deficiency.

This study was presented as poster presentation (poster number P_11) in 4^th^ International Workshop on HIV Pediatrics, 20 – 21 July 2012, Washington DC, USA.

This is a sub-study of the Pediatric Randomized Early versus Deferred Initiation in Cambodia and Thailand study (The PREDICT study, clinicaltrials.gov identification number NCT00234091).

## Competing interest

All authors declare no conflict of interest and that member of their immediate families do not have a financial interest in or arrangement with any commercial organization that may have a direct interest in the subject matter of this article.

## Authors’ contribution

TB, MI, YI, and WS were involved in the study design, collection of data, and writing of the manuscript. KM, TP, JA, AM, and SV were involved in the study design and collection of data. TN was involved in the study design. PP was involved in the study design and writing of the manuscript. JS and TB analyzed the data and wrote the manuscript. TI collected the data and assisted in the writing of the manuscript. All authors reviewed the draft of the manuscript before submission. All authors have read and approved the final manuscript.

## Authors information

CIP TH001:HIV Netherlands Australia Thailand (HIV-NAT) Research Collaboration, Thai Red Cross AIDS Research Center, Bangkok, Thailand; Dr.Kiat Ruxrungtham, Dr.Jintanat Ananworanich, Dr.Thanyawee Puthanakit, Dr.Chitsanu Pancharoen, Dr.Torsak Bunupuradah, Stephen Kerr, Theshinee Chuenyam,Sasiwimol Ubolyam, Apicha Mahanontharit,Tulathip Suwanlerk,Jintana Intasan,Thidarat Jupimai,Primwichaya Intakan, Tawan Hirunyanulux, Praneet Pinklow, Kanchana Pruksakaew,Oratai Butterworth, Nitiya Chomchey,Chulalak Sriheara,Anuntaya Uanithirat,Sunate Posyauattanakul,Thipsiri Prungsin,Pitch Boonrak,Waraporn Sakornjun, Tanakorn Apornpong,Jiratchaya Sophonphan,OrmrudeeRit-im,Nuchapong Noumtong,Noppong Hirunwadee,Dr.Chaiwat Ungsedhapand,Chowalit Phadungphon,Wanchai Thongsee,Orathai Chaiya,Augchara Suwannawat,Threepol Sattong,Niti Wongthai,Kesdao Nantapisan,Umpaporn Methanggool,Narumon Suebsri,Dr.Chris Duncombe,Taksin Panpuy,Chayapa Phasomsap,Boonjit Deeaium,Pattiya Jootakarn

CIP TH003:Bamrasnaradura Infectious Diseases Institute, Nonthaburi,Thailand; Dr.Jurai Wongsawat,Dr.Rujanee Sunthornkachit, Dr.Visal Moolasart,Dr.Natawan Siripongpreeda,Supeda Thongyen,Piyawadee Chathaisong,Vilaiwan Prommool, Duangmanee Suwannamass,Simakan Waradejwinyoo,Nareopak Boonyarittipat,Thaniya Chiewcharn,Sirirat Likanonsakul,Chatiya Athichathana, Boonchuay Eampokalap,Wattana Sanchiem.

CIP TH004:Srinagarind Hospital,Khon Kaen University,Khon Kaen,Thailand; Dr.Pope Kosalaraksa,Dr.Pagakrong Lumbiganon, Dr.Chulapan Engchanil,Piangjit Tharnprisan,Chanasda Sopharak,Viraphong Lulitanond,Samrit Khahmahpahte,Ratthanant Kaewmart, Prajuab Chaimanee, Mathurot Sala, Thaniita Udompanit,Ratchadaporn Wisai,Somjai Rattanamanee, Yingrit Chantarasuk,Sompong Sarvok,Yotsombat Changtrakun,Soontorn Kunhasura, Sudthanom Kamollert

CIP TH005:Queen Savang Vadhana Memorial Hospital, Chonburi,Thailand; Dr. Wicharn Luesomboon, Dr.Pairuch Eiamapichart,Dr.Tanate Jadwattanakul,Isara Limpet-ngam,Daovadee Naraporn,Pornpen Mathajittiphun,Chatchadha Sirimaskul,Woranun Klaihong,Pipat Sittisak,Tippawan Wongwian, Kansiri Charoenthammachoke,Pornchai Yodpo.

CIP TH007:Nakornping Hospital,ChiangMai,Thailand;Dr.Suparat Kanjanavanit, Dr.Maneerat Ananthanavanich, Dr.Penpak Sornchai,Thida Namwong,Duangrat Chutima,Suchitra Tangmankhongworakun,Pacharaporn Yingyong,Juree Kasinrerk, Montanee Raksasang,Pimporn Kongdong,Siripim Khampangkome, Suphanphilat Thong-Ngao,Sangwan Paengta,Kasinee Junsom, Ruttana KhuankaewM,Parichat Moolsombat,Duanpen Khuttiwung,Chanannat Chanrin.

CIP TH009:Chiangrai Regional Hopsital, ChiangRai, Thailand; Dr.Rawiwan Hansudewechakul,Dr.Yaowalak Jariyapongpaiboon, Dr.Chulapong Chanta,Areerat Khonponoi, Chaniporn Yodsuwan, WaruneeSrisuk,Pojjavitt Ussawawuthipong,Yupawan Thaweesombat,Polawat Tongsuk,Chaiporn Kumluang,Ruengrit Jinasen,Noodchanee Maneerat,Kajorndej Surapanichadul, Pornpinit Donkaew.

CIP TH010:National Pediatric Hospital,PhnomPenh,Cambodia;Dr.Saphonn Vonthanak, Dr. Ung Vibol,Dr.Sam Sophan,Dr.Pich Boren,Dr.Kea Chettra,Lim Phary,Toun Roeun, Tieng Sunly,Mom Chandara,Chuop Sokheng,Khin Sokoeun,Tuey Sotharin.

CIP TH011:Social Health Clinic,Phnom Penh,Cambodia;Dr.Saphonn Vonthanak,Dr.Ung Vibol,Dr.Vannary Bun,Dr.Somanythd Chhay Meng,Dr.Kea Chettra,Sam Phan,Wuddhika In vong,Khuon Dyna.

CIP TH012:PrapokklaoHospital,Chantaburi,Thailand;Dr.Chaiwat Ngampiyaskul,Dr.Naowarat Srisawat, Wanna Chamjamrat,Sayamol Wattanayothin,Pornphan Prasertphan,Tanyamon Wongcheeree, Pisut Greetanukroh,Chataporn Imubumroong, Pathanee Teirsonsern.
